# Antigenic sin of wild-type SARS-CoV-2 vaccine shapes poor cross-neutralization of BA.4/5/2.75 subvariants in BA.2 breakthrough infections

**DOI:** 10.1038/s41467-022-34400-8

**Published:** 2022-11-19

**Authors:** Bin Ju, Qing Fan, Miao Wang, Xuejiao Liao, Huimin Guo, Haiyan Wang, Xiangyang Ge, Lei Liu, Zheng Zhang

**Affiliations:** 1 Institute for Hepatology, National Clinical Research Center for Infectious Disease, Shenzhen Third People’s Hospital, Shenzhen, 518112 Guangdong Province China; 2 The Second Affiliated Hospital, School of Medicine, Southern University of Science and Technology, Shenzhen, 518112 Guangdong Province China; 3 Guangdong Key Laboratory for Anti-infection Drug Quality Evaluation, Shenzhen, 518112 Guangdong Province China; 4 Shenzhen Research Center for Communicable Disease Diagnosis and Treatment of Chinese Academy of Medical Science, Shenzhen, 518112 Guangdong Province China

**Keywords:** Immunology, Microbiology, SARS-CoV-2, Immune evasion

## Abstract

With declining SARS-CoV-2-specific antibody titers and increasing numbers of spike mutations, the ongoing emergence of Omicron subvariants causes serious challenges to current vaccination strategies. BA.2 breakthrough infections have occurred in people who have received the wild-type vaccines, including mRNA, inactivated, or recombinant protein vaccines. Here, we evaluate the antibody evasion of recently emerged subvariants BA.4/5 and BA.2.75 in two inactivated vaccine-immunized cohorts with BA.2 breakthrough infections. Compared with the neutralizing antibody titers against BA.2, marked reductions are observed against BA.2.75 in both 2-dose and 3-dose vaccine groups. In addition, although BA.2 breakthrough infections induce a certain cross-neutralization capacity against later Omicron subvariants, the original antigenic sin phenomenon largely limits the improvement of variant-specific antibody response. These findings suggest that BA.2 breakthrough infections seem unable to provide sufficient antibody protection against later subvariants such as BA.2.75 in the current immunization background with wild-type vaccines.

## Abstract (web summary)

Updated vaccines based on Omicron subvariants have started to being rolled out. Analysis of antibody response in individuals with two or three vaccine doses suggests that BA.2 breakthrough infection barely increases cross-neutralization capacity against BA.4/5 or BA.2.75.

## Introduction

During the coronavirus disease 2019 (COVID-19) pandemic worldwide, the continuous emergence of severe acute respiratory syndrome coronavirus 2 (SARS-CoV-2) variants have raised concerns about the immune escape and the effectiveness of available immunization strategy. Especially, the Omicron (B.1.1.529 or BA.1) variant emerged in November 2021 and then spread rapidly all over the world, harboring more than 30 amino acid mutations in the spike protein and strikingly escaping infection- and/or vaccination-elicited neutralizing antibodies (nAbs)^1–3^. With the continuing surveillance of SARS-CoV-2 evolution in the population, Omicron has been divided into a series of subvariants such as BA.2, BA.3, BA.4, and BA.5, also showing marked neutralization resistances to polyclonal plasma and monoclonal nAbs^4–7^. Meanwhile, the current SARS-CoV-2 infection status in people would also affect the protection effect against the later emerging variants. The Omicron BA.2 variant had ever dominated the COVID-19 wave in many countries and led to serious breakthrough infections among the vaccinated population, regardless of receiving mRNA, inactivated, or recombinant protein vaccines, etc.^8–11^.

Currently, BA.4 and BA.5 (hereafter, BA.4/5) sharing the same amino acid sequence of spike protein, have rapidly replaced BA.2 in South Africa and spread to other countries and regions with increasing global prevalence^9,12–14^. In addition, a recently emerging subvariant, BA.2.75, was first detected in India and rapidly growing in at least 15 countries, probably with more transmissibility than other BA.2 subvariants^11,15,16^. Both BA.4/5 and BA.2.75 emerged after the BA.2 and begun out competing with BA.2 globally. Several studies have demonstrated that BA.4/5 and BA.2.75 facilitate further escape from nAbs than BA.2^7,9,11^. However, it remains unclear if the current vaccination background in people would affect the effectiveness of next-generation vaccines against recently emerging Omicron subvariants, especially those designed based on the BA.2 variant.

In this work, we evaluate the cross-neutralization against pseudotyped BA.4/5 and BA.2.75 subvariants in the early stage of BA.2 breakthrough infections and during the convalescent period. Such laboratory evaluation is essential to guide future vaccine options and public health policy.

## Results

### Study design

**Fig. 1:**
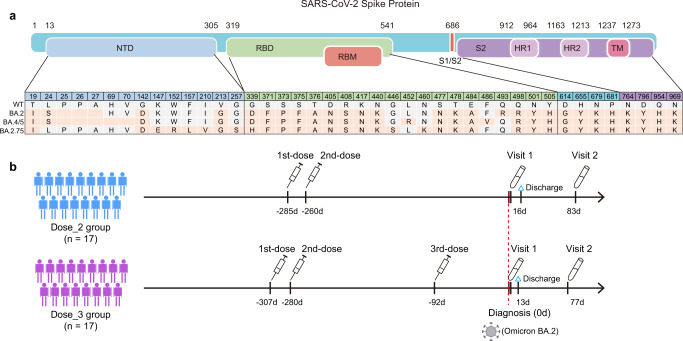
SARS-CoV-2 Omicron subvariants and BA.2 breakthrough infection cohorts involved in this study. **a** Mutations located in spike protein were identified in Omicron subvariants including BA.2, BA.4/5, and BA.2.75. Amino acid residues consistent with WT SARS-CoV-2 were marked in gray, and mutations in Omicron subvariants were marked in orange. **b** Schematic overview of study design. Blood samples from BA.2 breakthrough infected individuals previously immunized with 2-dose or 3-dose inactivated vaccines were collected in the early stage of infection (Visit 1, within 2 days after diagnosis) and during the convalescent period (Visit 2, 81 days post diagnosis).

Compared with BA.2, BA.4/5 carries 4 mutations in the spike protein (Del69-70, L452R, F486V, and R493Q), and BA.2.75 has 11 alterations (S24L, Ins25-27, K147E, W152R, F157L, I210V, G257S, D339H, G446S, N460K, and R493Q) (Fig. 1a). In this study, we constructed two pseudotyped BA.4/5 and BA.2.75 viruses based on our previous established methods for Pango lineage A (wild-type, WT) and BA.2^17–19^. Then, we measured the neutralizing antibody titers in plasma samples obtained from 34 individuals with BA.2 breakthrough infections (Fig. 1b, Supplementary Fig. 1, Supplementary Tables 1 and 2). All participants were confirmed as positive in RT-PCR test and admitted to Shenzhen Third People’s Hospital during the wave of BA.2 epidemic in Shenzhen in March, 2022. Seventeen had received two doses of SARS-CoV-2 inactivated vaccines and another 17 individuals received a homologous third booster vaccination. We collected their plasma samples in the very early stage of BA.2 infection (Visit 1, within 2 days after positive RT-PCR diagnosis) and during the convalescent period (Visit 2, 81 days post diagnosis). Therefore, a total of 68 plasma samples were evaluated for the neutralization against 4 types of SARS-CoV-2 pseudoviruses carrying different spike proteins of WT, BA.2, BA.4/5, and BA.2.75 subvariants, respectively (Supplementary Fig. 2 and Table 3). In this study, all mentioned neutralization results meant pseudotyped virus neutralization.

### Cross-neutralization against BA.4/5 and BA.2.75 subvariants in BA.2 breakthrough infections

**Fig. 2:**
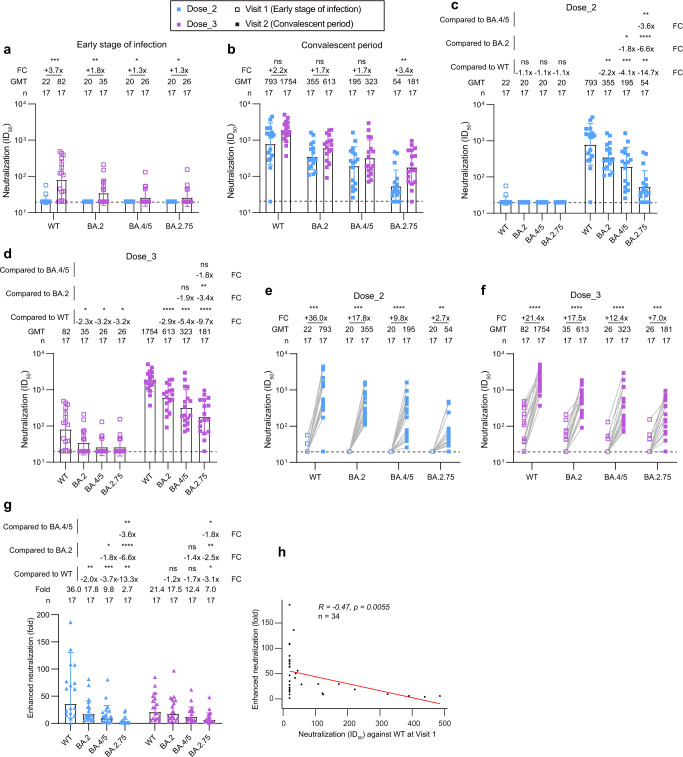
Neutralization of SARS-CoV-2 Omicron subvariants by plasma from inactivated vaccine recipients infected with BA.2. **a**, **b** Comparison of plasma neutralizing activities in different groups against WT SARS-CoV-2 and Omicron subvariants at Visit 1 (early stage of infection) (**a**) and Visit 2 (convalescent period) (**b**). **c**, **d** Neutralization titers against WT SARS-CoV-2 and Omicron subvariants in BA.2 breakthrough infected individuals with 2-dose (**c**) and 3-dose (**d**) inactivated vaccines. **e**, **f** Enhancement effects of neutralization against WT SARS-CoV-2 and Omicron subvariants by BA.2 breakthrough infection in 2-dose (**e**) and 3-dose (**f**) group. **g** Fold change in the enhanced neutralization of WT SARS-CoV-2 and Omicron subvariants by BA.2 breakthrough infection. **h** Correlation analysis between ID_50_ values against WT SARS-CoV-2 at Visit 1 and fold changes of enhanced neutralization in the 34 BA.2 breakthrough infected individuals. Perfect-fit correlation line was included on the plot. The non-parametric Spearman’s correlation coefficients (R) and statistically significant *P* value were provided. The ID_50_ values are means of at least two independent experiments. Data are presented as geometric mean values ± standard deviation (SD). The sample size, geometric mean, fold change, and significance of difference were labelled on the top. “-” represents decreased value and “+” represents increased value. Statistical significance was performed using two-tailed unpaired Wilcoxon test in (**a**, **b**), two-tailed Kruskal-Wallis test with paired Wilcoxon’s multiple-comparison test in (**c**, **d**, **g**), and two-tailed paired Wilcoxon test in (**e**, **f**). ****, *P* < 0.0001; ***, *P* < 0.001; **, *P* < 0.01; *, *P* < 0.05; ns, not significant. The dotted horizontal line in (**a-f**) indicates the limit of detection (1:20 dilution) for the neutralization assay. Non-neutralization data is set as 20 for analysis and visualization. ID_50_ indicates 50% inhibition dilution. GMT indicates geometric mean titer. FC indicates fold change. Source data and exact *P* values are provided as a Source Data file.

As shown in Fig. 2a, 2-dose inactivated vaccination established a low level of antibody memory against SARS-CoV-2 approximately 260 days post the last immunization. Only 11.8% (2/17) of plasma neutralized the WT virus, whose 50% inhibitory dilution (ID_50_) was more than the dilution of 1:20. All plasma lost their neutralizing activities against tested Omicron subvariants (BA.2, BA.4/5, and BA.2.75). By contrast, plasma in 3-dose group maintained relatively higher level of neutralization (70.6% of positive rate for WT, 41.2% for BA.2, 23.5% for BA.4/5, and 23.5% for BA.2.75). The neutralization of all plasma in 2-dose and 3-dose groups were rescued and enhanced by the BA.2 breakthrough infection (Fig. 2b). Of note, there were only slight differences in neutralizing activities against SARS-CoV-2 between 2-dose and 3-dose vaccination groups.

To better understand the differential antibody evasion of BA.4/5 and BA.2.75 subvariants, we rearranged these neutralization results by different groups to make a head-to-head comparison. As shown in Fig. 2c, in 2-dose breakthrough group, the geometric mean titers (GMTs) of plasma nAbs against BA.2, BA.4/5, and BA.2.75 were decreased by 2.2-fold, 4.1-fold, and 14.7-fold relative to that against WT. The GMTs against BA.4/5 and BA.2.75 were decreased by 1.8-fold and 6.6-fold relative to that against BA.2. Compared to BA.4/5, the GMT against BA.2.75 was significantly decreased by 3.6-fold. A similar downtrend was also observed in 3-dose breakthrough group (Fig. 2d), whose reductions were 2.9-fold (BA.2), 5.4-fold (BA.4/5), and 9.7-fold (BA.2.75) as compared to the WT. The GMT against BA.2.75 was also the lowest among all tested SARS-CoV-2 pseudoviruses. Together, these results demonstrated that the current immune barrier established by vaccination or vaccination plus the BA.2 breakthrough infection would be severely challenged by recently emerging Omicron subvariants.

Especially interesting, we noted that the enhancement effects of neutralization against WT SARS-CoV-2 and Omicron subvariants and between 2-dose and 3-dose groups are of significant difference. In 2-dose vaccinated individuals, the GMTs of plasma nAbs against WT, BA.2, BA.4/5, and BA.2.75 were increased by 36.0-fold, 17.8-fold, 9.8-fold, and 2.7-fold after the BA.2 breakthrough infection, respectively (Fig. 2e). By contrast, the increased folds of GMTs were 21.4-fold, 17.5-fold, 12.4-fold, and 7.0-fold against WT, BA.2, BA.4/5, and BA.2.75 in 3-dose vaccinated individuals, respectively (Fig. 2f). Finally, we analyzed the differential enhancement by the BA.2 breakthrough infection against WT SARS-CoV-2 and Omicron subvariants. As shown in Fig. 2g, the enhanced neutralization against WT was the largest in 2-dose breakthrough group, followed by BA.2, BA.4/5, and BA.2.75. However, this gap became obviously smaller in 3-dose breakthrough group. In addition, the enhancement of neutralizing activities against WT virus was negatively correlated to the ID_50_ values when the BA.2 infection established (Fig. 2h). Most of the plasma lost the neutralization against BA.2, BA.4/5, and BA.2.75 at Visit 1, whose ID_50_ values were uniformly set to 20 for analysis and visualization. So, similar correlation analysis was not performed for Omicron variants. These findings indicated that the BA.2 breakthrough infection mainly induced cross nAbs against the WT virus and poorly elicited Omicron-specific nAbs in prototype vaccine recipients.

## Discussion

SARS-CoV-2 specific memory B cells (MBCs) induced by previous vaccination could effectively respond to the subsequent BA.2 breakthrough infection, which was the source of increased cross-neutralization capacity. However, this recall of antibody memory was mainly due to the relative expansion of some MBCs recognizing the conserved epitopes between WT and variant. Those real BA.2-specific nAbs were much more difficult to be effectively induced in individuals who had received two or three doses of WT-inactivated vaccines. Similar finding was also clarified in BA.1 breakthrough infections^20,21^, demonstrating that BA.1-RBD-specific MBCs were very rare compared with WT-specific and WT/BA.1 cross-reactive MBCs in 2-dose and 3-dose mRNA-vaccinated individuals. Our evaluation focused on the enhancement effects of neutralization elicited by the BA.2 breakthrough infection benefiting from paired plasma collected in the very early stage of infection and during the convalescent period. The initial immune background could limit the antibody response induced by subsequent antigenic stimulation. This phenomenon is usually called as antigenic sin, which has been described in detail for influenza infections and vaccinations^22,23^. Together with the data from Quandt et al. and Kaku et al.^20,21,24^, we provided comprehensive evidences that original antigenic sin occurred in both Omicron BA.1 and BA.2 breakthrough infections.

Besides, three exposures to SARS-CoV-2 by vaccination and/or infection seemed to have led to the saturation of neutralizing antibody response. The fourth exposure by BA.2 breakthrough infection did not induce much higher titers of nAbs against WT, BA.2, and BA.4/5, which seemed to reach to “the ceiling effects”. Although BA.2 carried a total of 29 alterations in the spike protein compared with WT, it could be still regarded as a similar vaccine in terms of the full-length spike consisting of 1273 amino acids^4,25^. Our data revealed that further vaccination with similar vaccines might not benefit the public to combat the ongoing SARS-CoV-2 variants, especially recently emerging BA.4/5 and BA.2.75 subvariants, which showed significant antibody evasions in the current immune background. Thus, one of important direction for the updated vaccine is to design more unique immunogen to induce highly potent Omicron-specific nAbs. Those conserved epitopes between WT and Omicron variant may need to be abandoned, because our immune system has obtained sufficient antibody memory to them in the current immunization strategy and the titers of these cross nAbs could not be increased endlessly.

## Methods

### Study approval and plasma samples

This study was approved by the Ethics Committee of Shenzhen Third People’s Hospital, China (approval number: 2021-030). All participants had provided written informed consent for sample collection and subsequent analysis. All plasma samples were collected from 34 individuals (median age: 36, IQR: 21-64; male: 17, female: 17) infected with Omicron BA.2 subvariant in the early stage of breakthrough infection and during the period of convalescent, 17 (median age: 41, IQR: 22-64; male: 4, female: 13) of which were previously immunized by two doses of wild-type inactivated vaccines and another 17 participants (median age: 35, IQR: 21-51; male: 13, female: 4) had received a third homologous vaccine booster. Detailed information of participants was provided in Supplementary Tables 1 and 2. All participants involved in this study were local COVID-19 patients. According to the local policies, they were given free treatments and follow-up visits. The remaining plasma samples were stored at −80 °C in the Biobank of Shenzhen Third People’s Hospital and heat-inactivated at 56 °C for 1 h before use.

### SARS-CoV-2 pseudoviruses and neutralization assay

Pseudovirus was generated by co-transfection of HEK-293T cells (ATCC, CRL-3216) with a spike-expressing plasmid (WT, Pango lineage A (NC_045512), BA.2 (EPI_ISL_9652748), BA.4/5 (EPI_ISL_11542550), or BA.2.75 (EPI_ISL_13502576)) and an env-deficient HIV-1 backbone vector (pNL4-3.Luc.R-E-) (NIH AIDS Reagent Program, 3418)^17,18,26^. After two days, the culture supernatant was harvested, clarified by centrifugation, filtered, and stored at −80 °C before use. The infectious titer of each pseudovirus was measured by luciferase activity in the HEK-293T-hACE2 cells (YEASEN Biotech, 41107ES03) using Bright-Lite Luciferase reagent (Vazyme Biotech, DD1204-03). Detailed sequence information of each spike protein was shown in Fig. 1a. To determine the neutralizing activity, plasma samples were serially diluted and incubated with an equal volume of pseudovirus at 37 °C for 1 h. HEK-293T-hACE2 cells were subsequently added into 96-well plates. After an incubation of 48 h, the culture medium was removed and 100 μL of Bright-Lite Luciferase reagent was added. After 2 mins at RT, 90 μL of cell lysate was transferred to 96-well white solid plates for measurements of luminescence using Varioskan™ LUX multimode microplate reader (Thermo Fisher Scientific). The 50% inhibitory dilution (ID_50_) for plasma was calculated using GraphPad Prism 8 software by log (inhibitor) vs. normalized response - Variable slope (four parameters) model. The cut-off value of neutralization was set as 1:20 dilution. Non-neutralizing data below the limit was set to 20 for the analysis and visualization.

### Statistics & reproducibility

No statistical method was used to predetermine the sample size. No data were excluded from the analyses. The experiments were not randomized. The Investigators were not blinded to allocation during experiments and outcome assessment. Statistical significance was performed with two-tailed unpaired or paired Wilcoxon test or two-tailed Kruskal-Wallis test with paired Wilcoxon’s multiple-comparison test using R 4.1.3 software. The correlation analysis was performed using R 4.1.3 software. The non-parametric Spearman’s correlation coefficients (R) and statistically significant *P* value were provided. Symbol ‘*’ means *P* < 0.05, ‘**’ means *P* < 0.01, ‘***’ means *P* < 0.001, and ‘****’ means *P* < 0.0001. ns, not significant. All neutralization assays were performed at least two times independently.

## Supplementary information

supplementary information

reporting-summary

## Data Availability

The data generated in this study are provided in the Supplementary Information/Source Data file. Source data are provided with this paper.

## Source data

Source Data
