# Supplementary material for: Antigenic sin of wild-type SARS-CoV-2 vaccine shapes poor cross-neutralization of BA.4/5/2.75 subvariants in BA.2 breakthrough infections
Source: Nat Commun. 2022 Nov 19;13:7120. doi: 10.1038/s41467-022-34400-8 (PMC9675777; doi:10.1038/s41467-022-34400-8)
Supplement: Supplementary file 1 — supplementary information [file 41467_2022_34400_MOESM1_ESM.pdf]

Supplementary information

Antigenic sin of wild-type SARS-CoV-2 vaccine shapes poor cross-neutralization of BA.4/5/2.75 subvariants in BA.2 breakthrough infections

Bin Ju[1,2,3,*,#], Qing Fan[1,2,*], Miao Wang[1,2,*], Xuejiao Liao[1,2], Huimin Guo[1,2], Haiyan Wang[1,2], Xiangyang Ge[1,2], Lei Liu[1,2], Zheng Zhang[1,2,3,4,#]

[1]Institute for Hepatology, National Clinical Research Center for Infectious Disease, Shenzhen Third People's Hospital, Shenzhen 518112, Guangdong Province, China.

[2]The Second Affiliated Hospital, School of Medicine, Southern University of Science and Technology, Shenzhen 518112, Guangdong Province, China.

[3]Guangdong Key Laboratory for Anti-infection Drug Quality Evaluation, Shenzhen 518112, Guangdong Province, China.

[4]Shenzhen Research Center for Communicable Disease Diagnosis and Treatment of Chinese Academy of Medical Science, Shenzhen 518112, Guangdong Province, China.

*These authors contributed equally: Bin Ju, Qing Fan, and Miao Wang.
[#]Correspondence:
zhangzheng1975@aliyun.com (Zheng Zhang)
jubin2013@163.com (Bin Ju)

Supplementary information
Supplementary Figures 1-2
Supplementary Tables 1-3

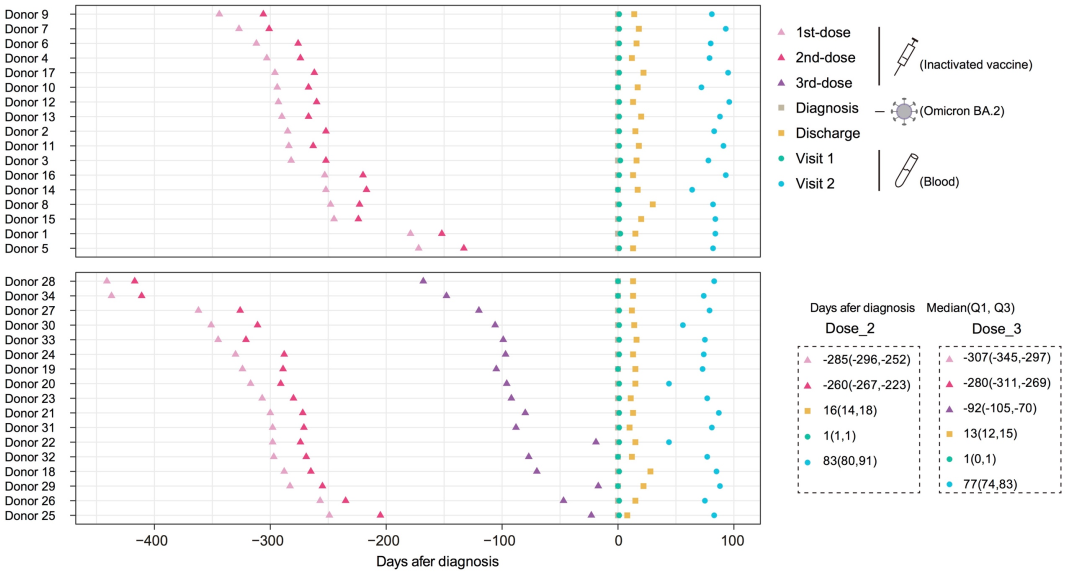

Supplementary Fig. 1 Timeline of events during wild-type SARS-CoV-2 inactivated vaccination, BA.2 breakthrough infection, and follow-up visit. Key events included two or three doses of inactivated vaccines, diagnosis, discharge, and two follow-up visits. The diagnosis date was normalized to Day 0 and the median time (IQR) to each stage marked on the right. The green and blue dots indicate blood samples collected at Visit 1 and Visit 2, respectively. Source data are provided as a Source Data file.

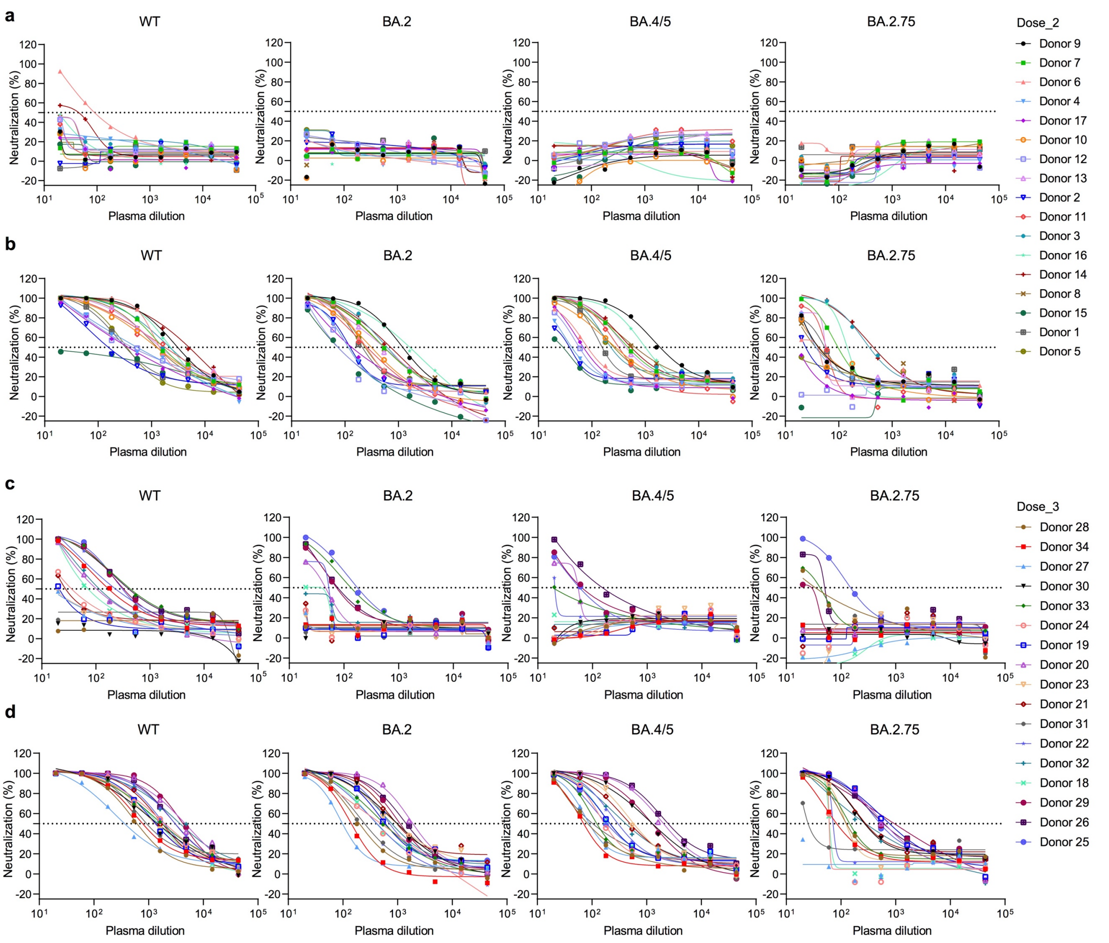

Supplementary Fig. 2 The plasma neutralization of 2-dose individuals (n = 17) at Visit 1 (a) and Visit 2 (b), and 3-dose individuals (n = 17) at Visit 1 (c) and Visit 2 (d) against the WT SARS-CoV-2 and Omicron subvariants. One representative curve from at least two independent experiments was displayed. A cut-off value of 50% is indicated by the dotted horizontal line in neutralization. Source data are provided as a Source Data file.

Supplementary Table 1 Detailed information of study subjects.

Donor	Gender	Age (range)	Group	1st-dose vaccination		2nd-dose vaccination		3rd-dose vaccination		Diagnosis	Discharge	Blood sampling (Visit 1 and Visit 2)	
				Date	Vaccine	Date	Vaccine	Date	Vaccine			Early stage of infection	Convalescent period
Donor 1	Female	61-70	Dose_2	2021/9/20	CoronaVac	2021/10/17	CoronaVac	/	/	2022/3/18	2022/4/2	2022/3/20	2022/6/10
Donor 2	Male	31-40	Dose_2	2021/6/5	CoronaVac	2021/7/8	CoronaVac	/	/	2022/3/17	2022/4/1	2022/3/18	2022/6/8
Donor 3	Male	41-50	Dose_2	2021/6/8	CoronaVac	2021/7/8	CoronaVac	/	/	2022/3/17	2022/4/2	2022/3/19	2022/6/3
Donor 4	Female	41-50	Dose_2	2021/5/12	CoronaVac	2021/6/10	CoronaVac	/	/	2022/3/11	2022/3/23	2022/3/12	2022/5/29
Donor 5	Female	21-30	Dose_2	2021/9/25	CoronaVac	2021/11/3	CoronaVac	/	/	2022/3/16	2022/3/29	2022/3/17	2022/6/6
Donor 6	Male	51-60	Dose_2	2021/4/30	CoronaVac	2021/6/5	CoronaVac	/	/	2022/3/8	2022/3/24	2022/3/9	2022/5/27
Donor 7	Female	31-40	Dose_2	2021/4/23	CoronaVac	2021/5/19	CoronaVac	/	/	2022/3/16	2022/4/3	2022/3/17	2022/6/17
Donor 8	Female	21-30	Dose_2	2021/7/8	CoronaVac	2021/8/2	CoronaVac	/	/	2022/3/13	2022/4/12	2022/3/14	2022/6/3
Donor 9	Male	21-30	Dose_2	2021/4/2	BBIBP-CorV	2021/5/10	BBIBP-CorV	/	/	2022/3/12	2022/3/26	2022/3/13	2022/6/1
Donor 10	Female	31-40	Dose_2	2021/5/28	BBIBP-CorV	2021/6/24	BBIBP-CorV	/	/	2022/3/18	2022/4/4	2022/3/18	2022/5/29
Donor 11	Female	21-30	Dose_2	2021/6/5	BBIBP-CorV	2021/6/26	BBIBP-CorV	/	/	2022/3/16	2022/4/3	2022/3/17	2022/6/15
Donor 12	Female	41-50	Dose_2	2021/5/22	BBIBP-CorV	2021/6/24	BBIBP-CorV	/	/	2022/3/11	2022/3/24	2022/3/12	2022/6/15
Donor 13	Female	41-50	Dose_2	2021/5/26	CoronaVac	2021/6/18	CoronaVac	/	/	2022/3/12	2022/4/1	2022/3/13	2022/6/8
Donor 14	Female	61-70	Dose_2	2021/7/4	BBIBP-CorV	2021/8/8	CoronaVac	/	/	2022/3/13	2022/3/30	2022/3/13	2022/5/16
Donor 15	Female	61-70	Dose_2	2021/7/18	BBIBP-CorV	2021/8/8	BBIBP-CorV	/	/	2022/3/20	2022/4/9	2022/3/21	2022/6/12
Donor 16	Female	61-70	Dose_2	2021/7/1	KCONVAC	2021/8/3	KCONVAC	/	/	2022/3/11	2022/3/24	2022/3/12	2022/6/12
Donor 17	Female	21-30	Dose_2	2021/5/22	WIBP-CroV	2021/6/25	WIBP-CroV	/	/	2022/3/14	2022/4/5	2022/3/15	2022/6/17
Donor 18	Male	51-60	Dose_3	2021/5/31	CoronaVac	2021/6/23	CoronaVac	2022/1/4	CoronaVac	2022/3/15	2022/4/12	2022/3/16	2022/6/8
Donor 19	Male	31-40	Dose_3	2021/4/27	CoronaVac	2021/6/1	CoronaVac	2021/12/2	CoronaVac	2022/3/17	2022/4/1	2022/3/17	2022/5/29
Donor 20	Male	31-40	Dose_3	2021/5/17	CoronaVac	2021/6/12	CoronaVac	2021/12/24	CoronaVac	2022/3/30	2022/4/14	2022/3/31	2022/5/13
Donor 21	Male	31-40	Dose_3	2021/5/19	CoronaVac	2021/6/16	CoronaVac	2021/12/25	CoronaVac	2022/3/15	2022/3/28	2022/3/16	2022/6/10
Donor 22	Female	41-50	Dose_3	2021/6/5	CoronaVac	2021/6/29	CoronaVac	2022/3/11	CoronaVac	2022/3/30	2022/4/14	2022/3/31	2022/5/13
Donor 23	Male	21-30	Dose_3	2021/5/20	CoronaVac	2021/6/16	CoronaVac	2021/12/21	CoronaVac	2022/3/23	2022/4/3	2022/3/24	2022/6/8
Donor 24	Female	31-40	Dose_3	2021/4/20	CoronaVac	2021/6/1	CoronaVac	2021/12/9	CoronaVac	2022/3/16	2022/3/29	2022/3/17	2022/5/29
Donor 25	Male	21-30	Dose_3	2021/7/22	CoronaVac	2021/9/4	CoronaVac	2022/3/5	CoronaVac	2022/3/28	2022/4/5	2022/3/29	2022/6/19
Donor 26	Male	31-40	Dose_3	2021/7/1	BBIBP-CorV	2021/7/23	CoronaVac	2022/1/27	CoronaVac	2022/3/15	2022/3/30	2022/3/16	2022/5/29
Donor 27	Female	21-30	Dose_3	2021/3/15	BBIBP-CorV	2021/4/20	BBIBP-CorV	2021/11/12	BBIBP-CorV	2022/3/12	2022/3/24	2022/3/13	2022/5/30
Donor 28	Male	31-40	Dose_3	2020/12/31	BBIBP-CorV	2021/1/24	BBIBP-CorV	2021/9/30	BBIBP-CorV	2022/3/17	2022/3/30	2022/3/17	2022/6/8
Donor 29	Male	31-40	Dose_3	2021/6/2	KCONVAC	2021/6/30	KCONVAC	2022/2/23	KCONVAC	2022/3/12	2022/4/3	2022/3/12	2022/6/8
Donor 30	Male	41-50	Dose_3	2021/4/4	BBIBP-CorV	2021/5/14	BBIBP-CorV	2021/12/5	BBIBP-CorV	2022/3/21	2022/4/4	2022/3/22	2022/5/16
Donor 31	Male	31-40	Dose_3	2021/5/23	BBIBP-CorV	2021/6/19	BBIBP-CorV	2021/12/19	BBIBP-CorV	2022/3/17	2022/3/27	2022/3/18	2022/6/6
Donor 32	Female	41-50	Dose_3	2021/5/23	BBIBP-CorV	2021/6/20	BBIBP-CorV	2021/12/29	BBIBP-CorV	2022/3/16	2022/3/28	2022/3/16	2022/6/1
Donor 33	Male	31-40	Dose_3	2021/4/9	BBIBP-CorV	2021/5/3	BBIBP-CorV	2021/12/11	BBIBP-CorV	2022/3/20	2022/4/5	2022/3/21	2022/6/3
Donor 34	Male	31-40	Dose_3	2021/1/3	BBIBP-CorV	2021/1/29	BBIBP-CorV	2021/10/19	BBIBP-CorV	2022/3/16	2022/3/29	2022/3/16	2022/5/29

Supplementary Table 2 Summary demographic characteristics of SARS-CoV-2 BA.2 breakthrough infected patients in this study.

	Dose_2<br>(n = 17)	Dose_3<br>(n = 17)	Total<br>(n = 34)	P values#<br>Dose_2 vs Dose_3
Median age (range)	41(22-64)	35(21-51)	36(21-64)	0.546
Male(n, %)	4(23.53)	13(76.47)	17(50.00)	0.0025
1st-dose to diagnosis, (median, IQR)	-285(-296,-252)	-307(-345,-297)	-296.5(-322.2,-282.2)	0.0072
2nd-dose to diagnosis, (median, IQR)	-260(-267,-223)	-280(-311,-269)	-268(-288.8,-252)	0.0062
3rd-dose to diagnosis, (median, IQR)	-	-92(-105,-70)	-92(-105,-70)	-
Discharge to diagnosis, (median, IQR)	16(14,18)	13(12,15)	15(13,17)	0.024
Visit 1 to diagnosis, (median, IQR)	1(1,1)	1(0,1)	1(1,1)	0.0982
Visit 2 to diagnosis, (median, IQR)	83(80,91)	77(74,83)	81(75,84.75)	0.0151
1st-dose to 2nd-dose, (median, IQR)	30(26,34)	27(24,35)	28(25.25,34.75)	0.743
2nd-dose to 3rd-dose, (median, IQR)	-	195(188,222)	195(188,222)	-

Statistical significance was determined using two-tailed unpaired Wilcoxon test. IQR, interquartile range.

Supplementary Table 3 The plasma neutralization of BA.2 breakthrough infected patients against the WT SARS-CoV-2 and Omicron subvariants.

Group	Donor	Visit 1 (Early stage of infection)				Visit 2 (Convalescent period)			
		WT	BA.2	BA.4/5	BA.2.75	WT	BA.2	BA.4/5	BA.2.75
Dose_2	Donor 1	20	20	20	20	306	291	177	50
	Donor 2	20	20	20	20	174	189	39	37
	Donor 3	20	20	20	20	2179	783	256	434
	Donor 4	20	20	20	20	556	115	43	41
	Donor 5	20	20	20	20	284	291	269	20
	Donor 6	57	20	20	20	1639	235	78	29
	Donor 7	20	20	20	20	1398	599	461	87
	Donor 8	20	20	20	20	1263	571	600	31
	Donor 9	20	20	20	20	3723	1402	1613	39
	Donor 10	20	20	20	20	1283	281	238	36
	Donor 11	20	20	20	20	1483	308	298	60
	Donor 12	20	20	20	20	378	132	51	20
	Donor 13	20	20	20	20	1544	457	410	68
	Donor 14	33	20	20	20	4489	876	506	481
	Donor 15	20	20	20	20	20	110	26	20
	Donor 16	20	20	20	20	2158	1622	954	240
	Donor 17	20	20	20	20	452	169	64	20
Dose_3	Donor 18	122	43	20	20	1348	424	197	70
	Donor 19	37	20	20	20	1531	615	195	961
	Donor 20	173	44	45	20	5074	1871	1919	522
	Donor 21	39	20	20	20	1965	987	468	354
	Donor 22	324	52	20	20	3049	494	348	99
	Donor 23	20	20	20	20	1385	871	575	149
	Donor 24	45	20	20	20	2509	1939	162	62
	Donor 25	439	210	53	155	1703	783	188	529
	Donor 26	486	76	131	44	2757	1555	2975	602
	Donor 27	20	20	20	20	367	91	103	20
	Donor 28	20	20	20	20	701	218	71	147
	Donor 29	222	70	54	45	4135	1406	1209	734
	Donor 30	20	20	20	20	1703	941	1239	368
	Donor 31	20	20	20	20	929	269	121	40
	Donor 32	109	20	20	20	3232	739	488	215
	Donor 33	390	150	20	58	2305	536	144	135
	Donor 34	124	20	20	20	1097	179	79	72

The ID_{50} values of each plasma were shown. The cut-off value of neutralization was set to 1:20 dilution. Non-neutralization data was set as 20 for analysis and visualization. The data are means of at least two independent experiments.
